# Heteroresistance to beta-lactam antibiotics may often be a stage in the progression to antibiotic resistance

**DOI:** 10.1371/journal.pbio.3001346

**Published:** 2021-07-20

**Authors:** Victor I. Band, David S. Weiss

**Affiliations:** 1 Emory Antibiotic Resistance Center, Atlanta, Georgia, United States of America; 2 Department of Microbiology and Immunology, Emory University, Atlanta, Georgia, United States of America; 3 Emory Vaccine Center, Atlanta, Georgia, United States of America; 4 Division of Infectious Diseases, Department of Medicine, Emory University School of Medicine, Atlanta, Georgia, United States of America; 5 Research Service, Atlanta VA Medical Center, Decatur, Georgia, United States of America; Universitat zu Koln, GERMANY

## Abstract

Antibiotic resistance is a growing crisis that threatens many aspects of modern healthcare. Dogma is that resistance often develops due to acquisition of a resistance gene or mutation and that when this occurs, all the cells in the bacterial population are phenotypically resistant. In contrast, heteroresistance (HR) is a form of antibiotic resistance where only a subset of cells within a bacterial population are resistant to a given drug. These resistant cells can rapidly replicate in the presence of the antibiotic and cause treatment failures. If and how HR and resistance are related is unclear. Using carbapenem-resistant Enterobacterales (CRE), we provide evidence that HR to beta-lactams develops over years of antibiotic usage and that it is gradually supplanted by resistance. This suggests the possibility that HR may often develop before resistance and frequently be a stage in its progression, potentially representing a major shift in our understanding of the evolution of antibiotic resistance.

## Abstract 2

A study of heteroresistance to broad range of beta-lactam antibiotics in clinical isolates of E. coli suggests that it may be an intermediate stage in the development of full antibiotic resistance, representing a shift in our understanding of the evolution of antibiotic resistance.

## Introduction

Heteroresistance (HR) is a poorly understood and underappreciated form of antibiotic resistance, wherein a bacterial strain contains a resistant subpopulation within a larger susceptible population [1]. This phenotypic form of resistance is distinct from conventional resistance in which all bacterial cells in a population display resistance to an antibiotic. Further, the resistant cells in a heteroresistant population can rapidly replicate in the presence of a given antibiotic, in contrast to persister cells, which are a distinct type of resistant subpopulation that is quiescent and cannot rapidly replicate when cultured with the drug [2]. Finally, while the resistant population in HR is enriched upon growth in a given antibiotic, the resistant cells reversibly return to baseline frequencies after subsequent drug-free subculture, indicating that the resistant subpopulation are not stable mutants.

HR has been described to many antibiotics and among many bacterial species [1,3–5], although the in vivo relevance of the phenomenon has been unclear [6]. However, recent findings have demonstrated that HR can mediate in vivo treatment failure [7–9] even though the resistant subpopulation of cells makes up a minority of the total population. Additionally, HR isolates harboring a very low frequency of resistant cells (<1 in 10,000) are usually misclassified as antibiotic susceptible, which could lead to inappropriate therapy. The origins of HR are unclear, as is the relationship between HR and conventional resistance in which all the cells in a population are phenotypically resistant.

To gain insight into the relationship between HR and resistance, we assessed the proportion of isolates exhibiting these phenotypes to beta-lactam antibiotics among a pool of carbapenem-resistant Enterobacterales (CRE) from Georgia, USA area hospitals. This pool of isolates was collected from 2013 to 2015 as a part of the Multi-site Gram-negative Surveillance Initiative (MuGSI), with clear inclusion criteria [10] to ensure a representative sampling of hospital isolates.

## Results

**Fig 1:**
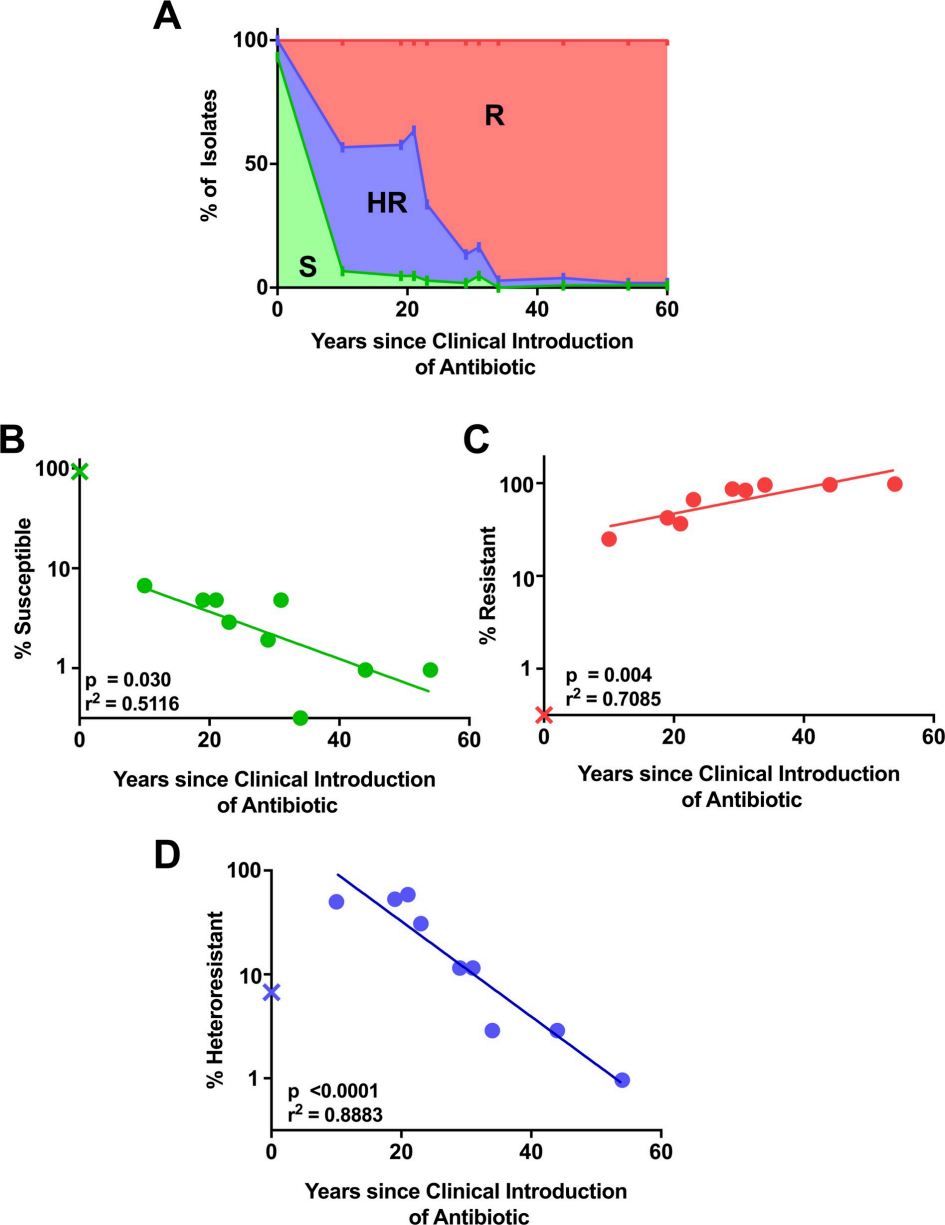
Incidence of heteroresistance and resistance correlates with number of years of clinical use of beta-lactam antibiotics. **(A)** A total of 104 isolates of CRE from Georgia area hospitals were assayed for heteroresistance, resistance, and susceptibility to 10 beta-lactam antibiotics that were introduced clinically in different years (ampicillin, 1961; cefazolin, 1971; amoxicillin/clavulanate, 1981; ceftazidime, 1984; aztreonam, 1986; piperacillin/tazobactam, 1992; cefepime, 1994; meropenem, 1996; doripenem, 2005; and ceftazidime/avibactam, 2015). Percent incidence of each designation was plotted by the age of the drug. Green area, susceptible (S); red area, resistant (R); blue area, heteroresistant (HR). (**B–D)** Incidence of (**B**) S, (**C**) R, and (**D**) HR isolates by clinical age of antibiotic. Linear regression on log-transformed data is shown with r-squared and *p*-value for slope significance. Ceftazidime/avibactam marked with “X” was introduced after the study isolates were collected and was not included in linear regression analysis. All data used to generate plots are available in S1 Data. CRE, carbapenem-resistant Enterobacterales; HR, heteroresistant; R, resistant; S, susceptible.

We used the population analysis profile (PAP) method to assess the presence of HR and detect resistant subpopulations of cells [9]. The PAP method consists of plating bacteria on solid media with a range of concentrations of antibiotic and assessing proportions of the bacterial population growing on each dose of drug. We tested 104 CRE from the Georgia MuGSI collection against 10 beta-lactam antibiotics that were first used clinically in the years ranging from 1961 (ampicillin) to 2015 (ceftazidime-avibactam). To minimize the chance of clinical samples containing 2 distinct bacterial isolates (mixed cultures), we tested each isolate after growth from a single colony. The resulting PAP curves for each isolate and antibiotic were classified as susceptible, resistant, or heteroresistant using previously determined criteria [8] and as described in the Materials and methods. Unsurprisingly, rates of susceptibility were highest for drugs with fewer years of clinical use, while the highest rates of resistance were observed for drugs with the most years of clinical use (Fig 1A). Accordingly, there was a significant negative correlation between the years since clinical introduction of an antibiotic and the proportion of susceptible isolates (Fig 1B), and a significant positive correlation with the proportion of resistant isolates (Fig 1C). In contrast, the proportion of isolates heteroresistant to an antibiotic was highest for the drugs with an intermediate number of years since clinical introduction; over 50% of isolates were HR to meropenem, doripenem, and cefepime, which have been in clinical use for 10 to 22 years (Fig 1A). This surprising trend suggests that after introduction of a new beta-lactam, there is an initial increase in the proportion of isolates exhibiting HR, a peak, and then a subsequent decrease, as HR is supplanted by resistance. Overall, there was a significant negative correlation between the proportion of heteroresistant isolates and the years since clinical introduction of the drug (Fig 1D).

Among the 10 antibiotics tested, some were observed to be correlated for susceptibility, resistance, and HR (S2A, S2C, and S2E Fig). However, even when antibiotics were excluded until no such correlated antibiotics remained, the statistically significant trends of decreased HR and susceptibility and increased resistance over time were maintained (S2B, S2D, and S2F Fig). Additionally, altering the cutoffs of the definition for HR did not impact this trend. Inclusion of strains with resistant subpopulations only surviving at 1× the breakpoint but not at higher concentrations (modified HR), or strains with 100% survival at 1× the breakpoint but with a subpopulation surviving at 2× the breakpoint and higher (Hi-HR) (S3A Fig), still led to observation of the trend of decreased HR over time (S3B and S3C Fig).

**Fig 2:**
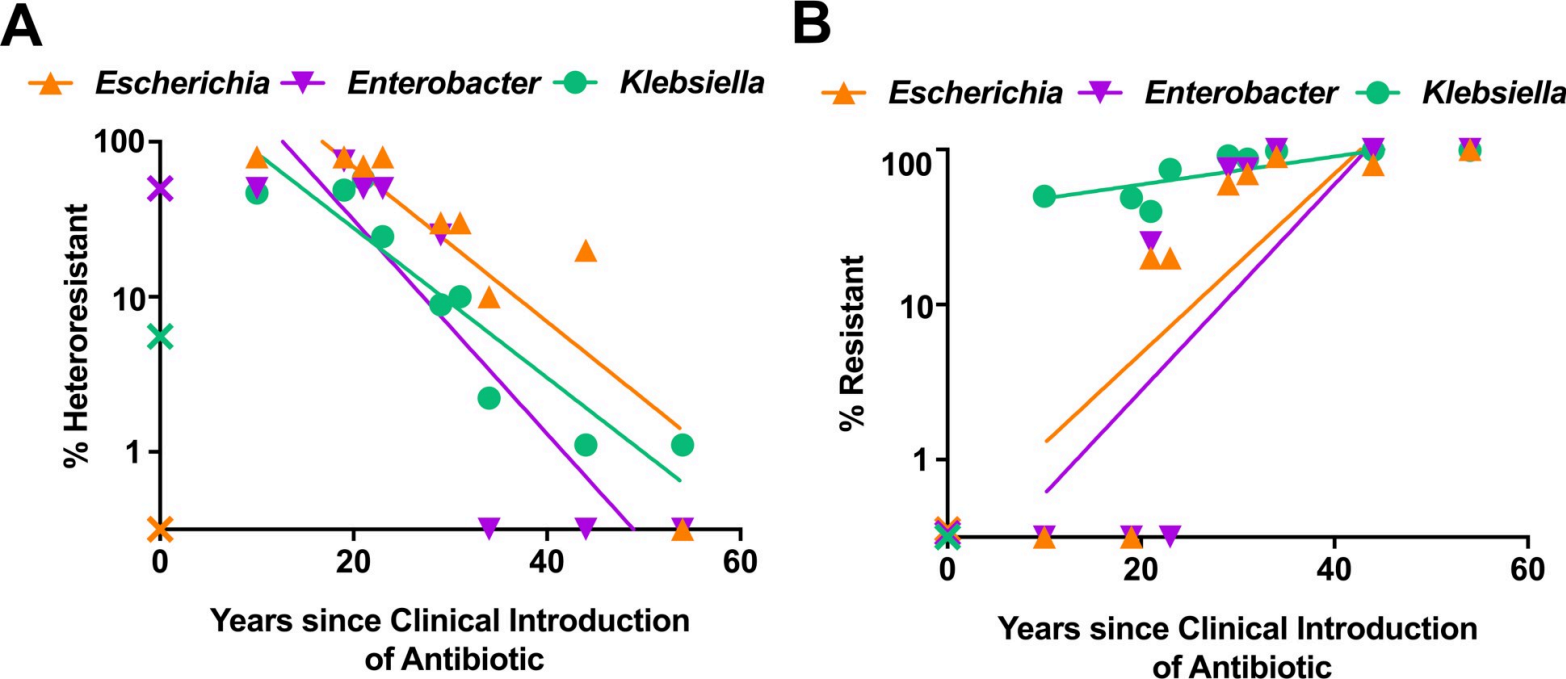
Incidence of resistance and heteroresistance by genus. **(A, B)** A total of 104 isolates of CRE from Georgia area hospitals were assayed for heteroresistance and resistance to 10 beta-lactam antibiotics and separated by genus: *Escherichia* (*n =* 10), *Enterobacter* (*n* = 4), and *Klebsiella* (*n* = 90). Percent incidence of heteroresistance (**A**) and resistance (**B**) among the 3 genera is shown. Linear regressions are shown with r-squared and *p*-values for non-zero slope as follows: (**A**, *Escherichia p* = 0.0023, r^2^ = 0.7577; *Enterobacter p* = 0.0047, r^2^ = 0.7608; *Klebsiella p* = 0.0003, r^2^ = 0.8584; **B**, *Escherichia p* = 0.0162, r^2^ = 0.5861; *Enterobacter p* = 0.0204, r^2^ = 0.5598; *Klebsiella p* = 0.0127, r^2^ = 0.6119). Ceftazidime/avibactam marked with “X” was introduced after the study isolates were collected and was not included in linear regression analysis. All data used to generate plots are available in S1 Data. CRE, carbapenem-resistant Enterobacterales.

When the Enterobacterales isolates were separated by genus, we observed similar patterns for each, where *Escherichia*, *Enterobacter*, and *Klebsiella* all displayed a significant negative correlation between incidence of HR and years of clinical antibiotic use (Fig 2A). A similar positive correlation was observed for all genera between incidence of resistance and years of clinical antibiotic use (Fig 2B). These data indicate that the HR phenomenon described here is not genus specific but rather that it occurs across all of the different Enterobacterales represented in this study.

## Discussion

Here, we used an incidence- and population-based approach to study resistance to 10 beta-lactams across clinical isolates of CRE from Georgia, USA. These isolates allow us to look at a snapshot of the collective resistance profile during the study years of 2013 to 2015. While we cannot track the development of resistance in individual isolates, we can observe proportions of resistance and consider the years of clinical use of drugs to estimate total antibiotic exposure. These results showed that for drugs in clinical use for the shortest amount of time, susceptibility dominated, while resistance dominated for those with the longest clinical use. Importantly, HR was most common to drugs introduced in the intermediate years, suggesting its role as a possible intermediate step in the development of resistance. In the future, our incidence-based study would be complemented by longitudinal studies that follow related isolates over time to observe the development of resistance in sets of related isolates. Additionally, confirmation of the role of HR in the progression of antibiotic resistance, and distinction between these pathways, will need to be explored experimentally with in vitro evolution experiments, which may prove challenging as the evolution of HR has not often been directly observed and the conditions under which this process occurs are relatively unclear.

**Fig 3:**
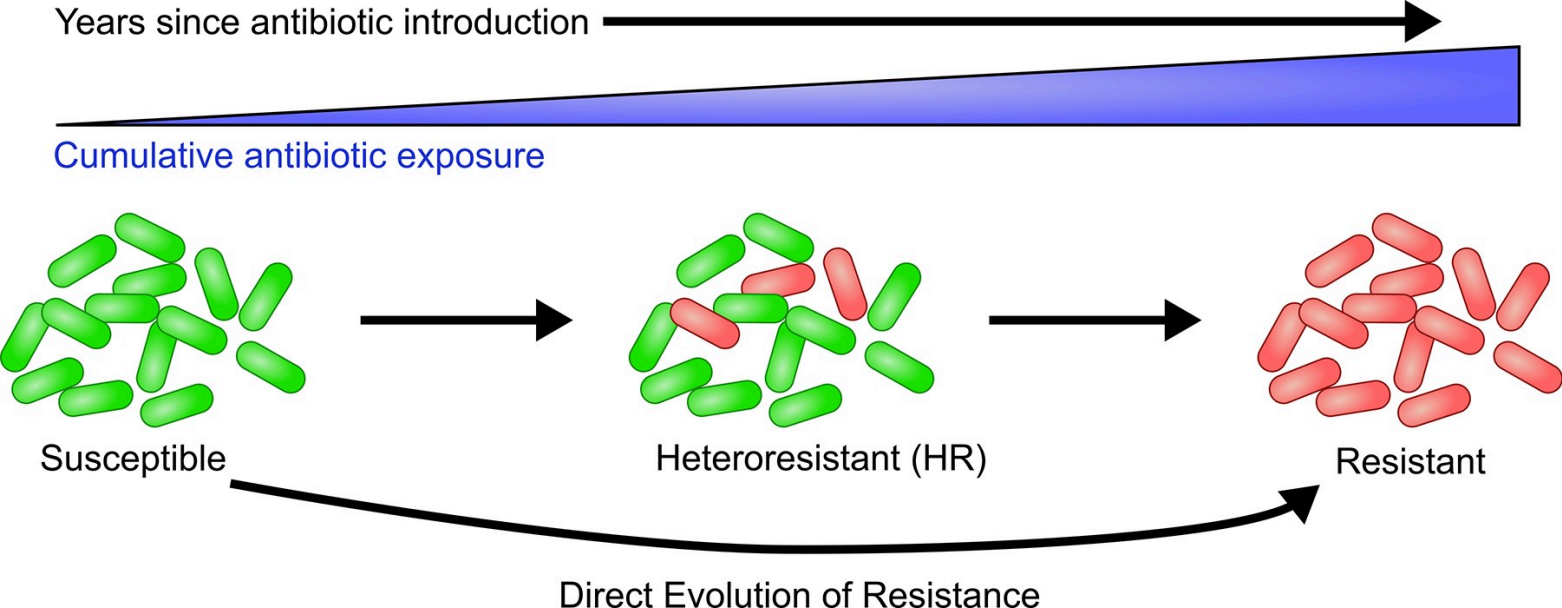
Proposed model for the evolution of resistance through heteroresistance. With increasing years after the introduction of an antibiotic, bacterial strains are cumulatively exposed to an increasing amount of the antibiotic. Strains are initially susceptible (green cells), but antibiotic exposure leads to the development of a resistant subpopulation (red cells). Increased antibiotic exposure eventually leads to resistance. Direct evolution of resistant isolates from susceptible isolates is also indicated. HR, heteroresistant.

These data have significant implications for our understanding of the development of antibiotic resistance, suggesting that HR could often be an evolutionary stage in the development of resistance (Fig 3). One weakness in this model is that only a single antibiotic from our analysis, ceftazidime/avibactam, suggests that HR evolves from susceptibility, and thus additional experimental evidence would be needed to further explore and potentially confirm this finding. This model would represent a major shift from the dogmatic pathway to resistance, in which a new resistance gene or accumulation of mutations is thought to be exhibited by all the cells in the population [11]. There are at least 2 obvious pathways to resistance that would involve HR either directly or indirectly. The first is that HR could be a direct stage in the progression to resistance, where the genetic changes causing HR are built upon, and after acquisition of further genetic changes, 100% of the cells in the population exhibit resistance. In such a scenario, reversal of the initial genetic changes causing HR would likely result in some of the cells in the resistant strain becoming susceptible, and thus the strain would appear HR once again. The second pathway is an indirect role for HR, where HR is an intermediate step that facilitates survival of sufficient cells in the total population, which then acquire distinct genetic changes that cause resistance. In this model, the genetic changes causing HR play a supportive role, and once resistance is achieved, their reversal would not have an impact on resistance. These direct and indirect roles for HR in the progression to resistance could each occur in different isolates. It is important to note that this study focuses only on beta-lactams and that the observed trends may not apply to other antibiotic classes and warrants further study. If HR is indeed often an initial evolutionary step toward resistance, it may then represent an important source of antibiotic resistance among clinical isolates.

Previous studies have demonstrated that roughly half of HR involves a low proportion of resistant cells and is generally undetected by current clinical susceptibility testing methods [7,9], suggesting that the initial steps foretelling the development of resistance may be missed. Supporting this concept is our finding that for the most recently introduced antibiotic in the study, ceftazidime/avibactam (introduced in 2015), we observed several heteroresistant isolates with low frequency resistant subpopulations but no isolates exhibiting resistance. This was the only data point contributing to this trend, and thus much more experimental evidence would be needed to confirm this hypothesis. However, if confirmed, low frequency and clinically undetected HR could be the initial harbinger of impending resistance that could be surveyed epidemiologically to inform public health and antibiotic prescribing practices.

## Materials and methods

### Sample collection

The 104 isolates used in this study were collected by the Georgia Emerging Infections Program (GA EIP), as a part of the Centers for Disease Control (CDC) MuGSI, detailed in Guh and colleagues [10]. A CRE case was defined as a carbapenem-nonsusceptible and extended-spectrum cephalosporin-resistant (ceftriaxone, ceftazidime, ceftizoxime, and cefotaxime) *Escherichia coli*, *Klebsiella aerogenes*, *Enterobacter cloacae* complex, *Klebsiella pneumoniae*, or *Klebsiella oxytoca* isolate recovered from a body site that is normally sterile or urine from individuals residing in the surveillance area during January 2013 to December 2015. Isolates were collected by participating Georgia hospitals and tested for inclusion criteria by GA EIP and CDC.

### Population analysis profile

PAP was performed as previously described [9]. Isolates were grown up from single colonies overnight and plated on 6 plates containing 0-, 0.25-, 0.5-, 1-, 2-, or 4-fold the breakpoint amount of antibiotic. Growth from single colonies minimizes the chances of assessing mixed cultures of bacteria that can be found in clinical isolates. The breakpoints used were defined by CLSI [12] as the minimum inhibitory concentration (MIC) for resistant isolates. Colonies were enumerated and survival was calculated by comparing to a drug-free plate. Isolates were classified as previously described [9] and as indicated in S1 Fig; susceptible if less than 1 in 10^−6^ survived at 1× the breakpoint, resistant if more than 50% survived at or above 1× the breakpoint, and heteroresistant if at least 1 in 10^−6^ but fewer than 50% of cells survived at 1× to 2× the breakpoint or above. Setting the threshold for HR at 1 in 10^−6^ makes the inadvertent inclusion of stable resistant mutants unlikely, since they would need to be present at a high level representing an extremely high mutation rate. Additionally, setting the cutoff for HR at 2× the breakpoint requires a resistant subpopulation for at least 2 datapoints, (1× and 2× breakpoint) and increases stringency for this definition. Each isolate was tested against 10 beta-lactam antibiotics: ampicillin, cefazolin, amoxicillin/clavulanate, ceftazidime, aztreonam, piperacillin/tazobactam, cefepime, meropenem, doripenem, and ceftazidime/avibactam. Raw CFU counts and interpretations are available in S1 Data.

### Data analysis

Year of clinical introduction for each antibiotic used in this study was determined by the approval date by the US Food and Drug Administration. Correlations with year of clinical introduction were calculated using a linear regression analysis, on data which was log transformed for percentage of isolates in each category. Correlations between antibiotics were calculated using the Cramer’s V method, on categorical PAP data that was one-hot encoded for each designation (susceptible, resistant, and heteroresistant). Best fit curves were calculated using a Gaussian distribution fit equation. All statistical calculations and regressions were done in Graphpad Prism 9.

## Supporting information

S1 Fig Designation of study strains by PAP. A representative graph of a susceptible (blue), heteroresistant (green), and resistant (orange) isolate from this study. Isolates are designated resistant if at 1× or above the breakpoint there is survival of at least 50% (0.5) of the population. If the isolate is not resistant, it is designated heteroresistant if at least 1 in 10^−6^ bacteria survive at 1× and 2× or above the breakpoint. If the isolate is neither resistant nor heteroresistant, it will fall below 10^−6^ surviving cells at or below the breakpoint and is designated susceptible. PAP, population analysis profile.
(TIFF)

S2 Fig Correlation analysis of antibiotics by PAP designation. Correlation analysis was performed on each antibiotic using the PAP designations generated in this study. (**A, C, E**) Matrix of the Cramer’s V statistic for the correlation of each antibiotic based on its categorical PAP designation. Antibiotic pairs were considered significantly correlated with each other when the Cramer’s V value had *p* < 0.05. * Stars indicate antibiotics that were removed from analysis in panels B, D, or F. (**B, D, F**) Percent incidence of each PAP designation was plotted by the age of the drug, excluding antibiotics that were significantly correlated with each other. For each pair of correlated antibiotics, one was excluded until there were no significant correlations remaining. Linear regression analysis with *p*-value and r-squared is indicated in each panel. All data used to generate plots are available in S1 Data. PAP, population analysis profile.
(TIFF)

S3 Fig Data comparison using different heteroresistance definitions. Heteroresistance definition as outlined in S1 Fig was altered to include/exclude distinct resistant subpopulations. (**A)** A representative graph of a susceptible (blue), HR (green), modified HR (aqua), Hi-HR (brown), and resistant (orange) isolate that fits each definition. Isolates are designated resistant if at 1× or above the breakpoint there is survival of at least 50% (0.5) of the population. If the isolate is not resistant, it will be designated HR if at least 1 in 10^−6^ bacteria survive at 1× and 2× above the breakpoint. If the isolate is neither resistant nor HR, it will be considered Hi-HR if at least 1 in 10^−6^ bacteria survive at 2× and 4× the breakpoint. In addition, isolates with at least 1 in 10^−6^ bacteria surviving at 1× the breakpoint with no requirement for subpopulations surviving at higher concentrations fit the definition for “modified HR.” Susceptible isolates will fall below 10^−6^ survival at or below the breakpoint. (**B–E)** Number of isolates designated as (**B**) HR, (**C**) HR (modified definition), or (**D**) Hi-HR based on PAP analysis using these new definitions. In (**E)**, isolates that fit any of the 3 HR definitions in **B–D** were included as HR (combined). A best fit curve was calculated for each using a Gaussian distribution equation. All data used to generate plots are available in S1 Data. BP, breakpoint; HR, heteroresistant.
(TIFF)

S1 Data Raw PAP data and designations used in analysis and figure preparation. PAP, population analysis profile.
(XLSX)

## Abbreviations

CDC: Centers for Disease Control and Prevention
CRE: carbapenem-resistant Enterobacterales
GA EIP: Georgia Emerging Infections Program
HR: heteroresistance
MIC: minimum inhibitory concentration
MuGSI: Multi-site Gram-negative Surveillance Initiative
PAP: population analysis profile

## References

[1] Band VI, Weiss DS. Heteroresistance: A cause of unexplained antibiotic treatment failure? PLoS Pathog. 2019;15(6). doi: 10.1371/journal.ppat.1007726 31170271 PMC6553791

[2] Brauner A, Fridman O, Gefen O, Balaban NQ. Distinguishing between resistance, tolerance and persistence to antibiotic treatment. Nat Rev Microbiol. 2016;14:320–30. doi: 10.1038/nrmicro.2016.34 27080241

[3] El-Halfawy OM, Valvano MA. Antimicrobial heteroresistance: an emerging field in need of clarity. Clin Microbiol Rev. 2015;28(1):191–207. doi: 10.1128/CMR.00058-14 25567227 PMC4284305

[4] Andersson DI, Nicoloff H, Hjort K. Mechanisms and clinical relevance of bacterial heteroresistance. Nat Rev Microbiol. 2019;17(8):479–96. doi: 10.1038/s41579-019-0218-1 31235888

[5] Nicoloff H, Hjort K, Levin BR, Andersson DI. The high prevalence of antibiotic heteroresistance in pathogenic bacteria is mainly caused by gene amplification. Nat Microbiol. 2019;4(3):504–14. doi: 10.1038/s41564-018-0342-0 30742072

[6] Falagas ME, Makris GC, Dimopoulos G, Matthaiou DK. Heteroresistance: a concern of increasing clinical significance? Clin Microbiol Infect. 2008;14(2):101–4. doi: 10.1111/j.1469-0691.2007.01912.x 18093235

[7] Band VI, Satola SW, Burd EM, Farley MM, Jacob JT, Weiss DS. Carbapenem-Resistant *Klebsiella pneumonia* Exhibiting Clinically Undetected Colistin Heteroresistance Leads to Treatment Failure in a Murine Model of Infection. mBio. 2018;9(2). 10.1128/mBio.02448-17 PMC5844991 29511071

[8] Band VI, Hufnagel DA, Jaggavarapu S, Sherman EX, Wozniak JE, Satola SW, et al. Antibiotic combinations that exploit heteroresistance to multiple drugs effectively control infection. Nat Microbiol. 2019;4(10):1627–35. doi: 10.1038/s41564-019-0480-z 31209306 PMC7205309

[9] Band VI, Crispell EK, Napier BA, Herrera CM, Tharp GK, Vavikolanu K, et al. Antibiotic failure mediated by a resistant subpopulation in Enterobacter cloacae. Nat Microbiol. 2016;1(6):16053. doi: 10.1038/nmicrobiol.2016.53 27572838 PMC5154748

[10] Guh AY, Bulens SN, Mu Y, Jacob JT, Reno J, Scott J, et al. Epidemiology of Carbapenem-Resistant Enterobacteriaceae in 7 US Communities, 2012–2013. JAMA. 2015;314(14):1479–87. doi: 10.1001/jama.2015.12480 26436831 PMC6492240

[11] Hughes D, Andersson DI. Environmental and genetic modulation of the phenotypic expression of antibiotic resistance. FEMS Microbiol Rev. 2017;41(3):374–91. doi: 10.1093/femsre/fux004 28333270 PMC5435765

[12] Clinical Laboratory and Standards Institute. Performance standards for antimicrobial susceptibility testing, 30th edition. 2020.

