# Supplementary figures and images for: Heteroresistance to beta-lactam antibiotics may often be a stage in the progression to antibiotic resistance
Source: PLoS Biol. 2021 Jul 20;19(7):e3001346. doi: 10.1371/journal.pbio.3001346 (PMC8351966; doi:10.1371/journal.pbio.3001346)

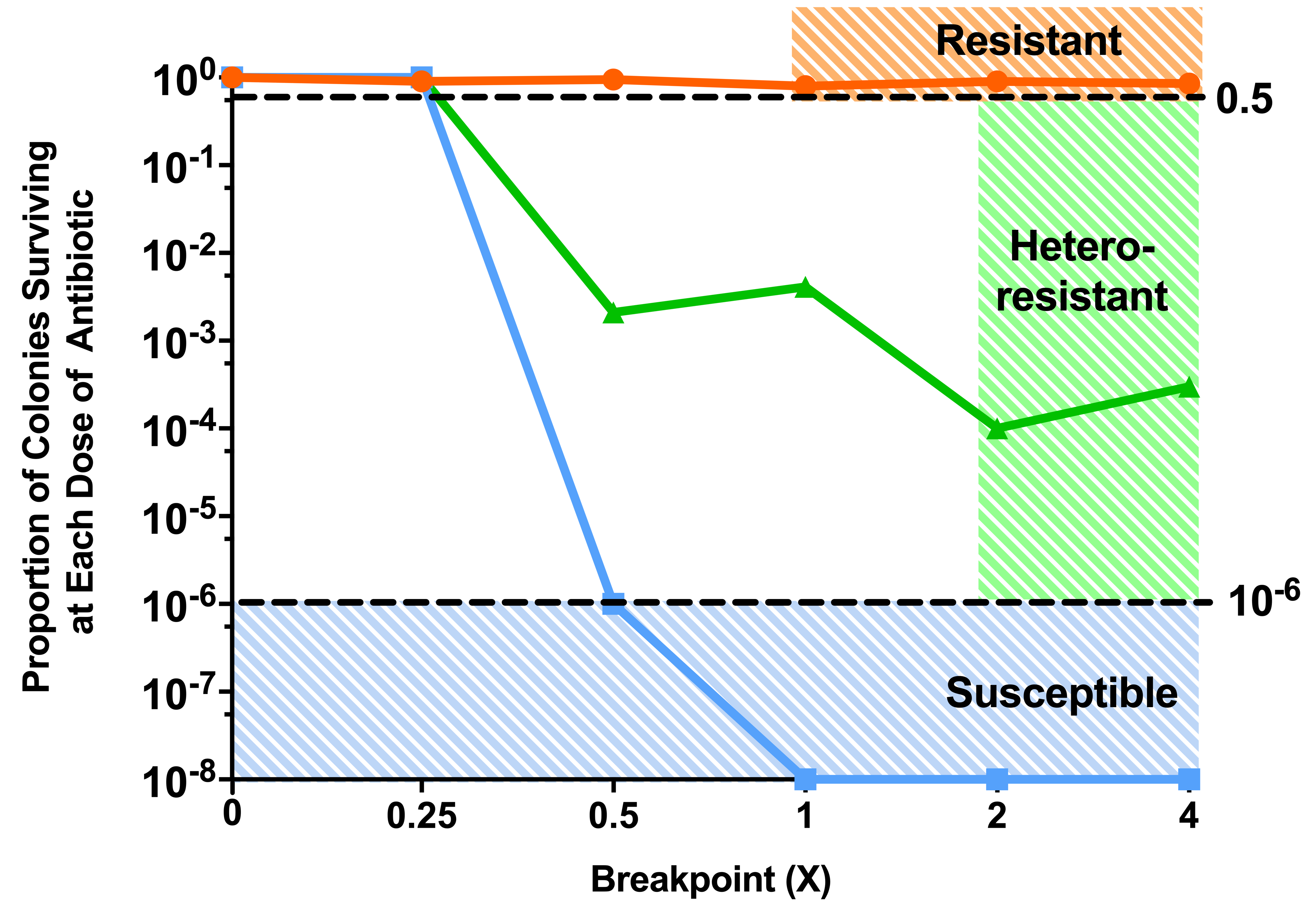

Supplement: S1 Fig — A representative graph of a susceptible (blue), heteroresistant (green), and resistant (orange) isolate from this study. Isolates are designated resistant if at 1× or above the breakpoint there is survival of at least 50% (0.5) of the population. If the isolate is not resistant, it is designated heteroresistant if at least 1 in 10−6 bacteria survive at 1× and 2× or above the breakpoint. If the isolate is neither resistant nor heteroresistant, it will fall below 10−6 surviving cells at or below the breakpoint and is designated susceptible. PAP, population analysis profile. (TIFF) [file pbio.3001346.s001.tiff]

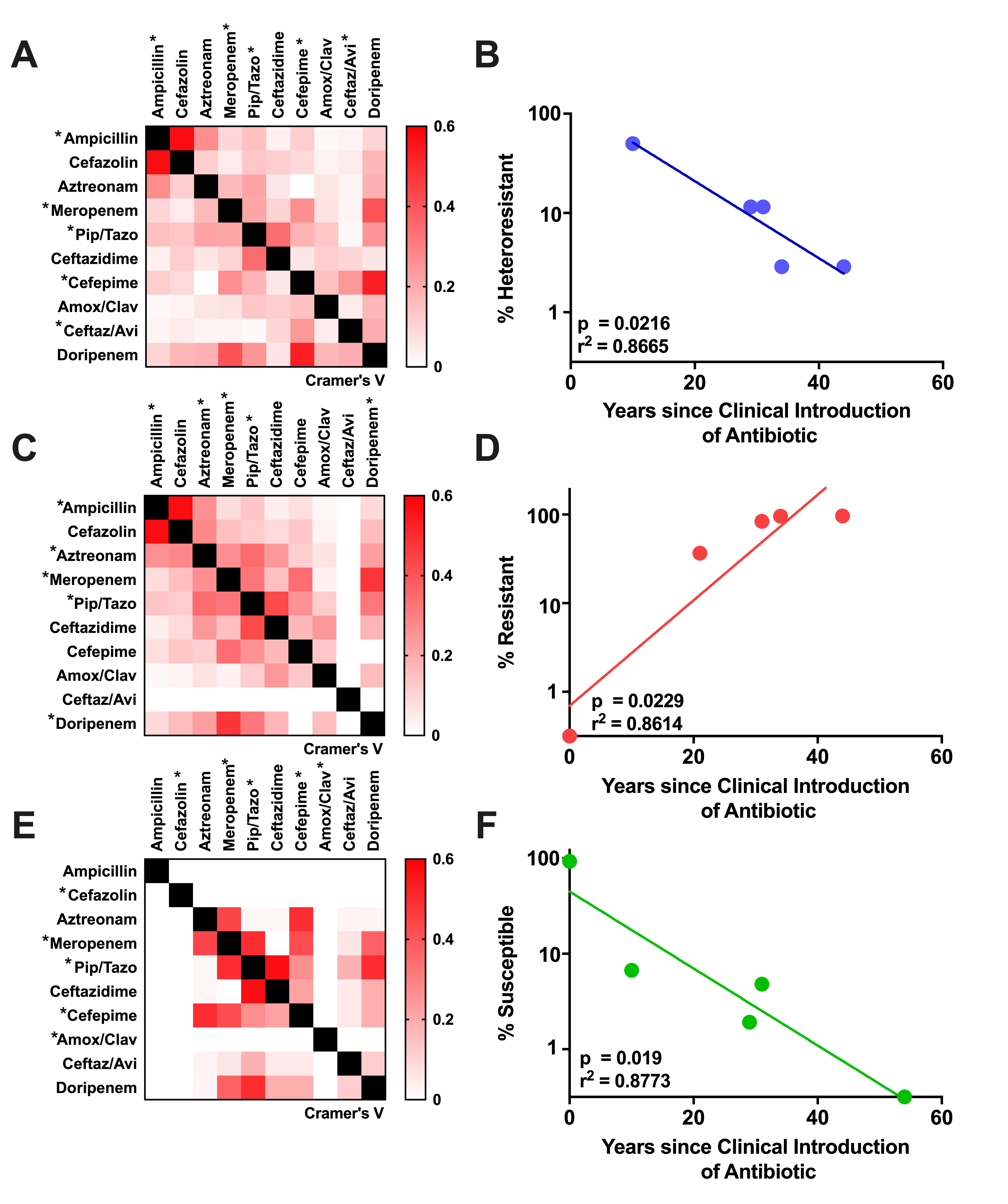

Supplement: S2 Fig — Correlation analysis was performed on each antibiotic using the PAP designations generated in this study. (A, C, E) Matrix of the Cramer’s V statistic for the correlation of each antibiotic based on its categorical PAP designation. Antibiotic pairs were considered significantly correlated with each other when the Cramer’s V value had p < 0.05. * Stars indicate antibiotics that were removed from analysis in panels B, D, or F. (B, D, F) Percent incidence of each PAP designation was plotted by the age of the drug, excluding antibiotics that were significantly correlated with each other. For each pair of correlated antibiotics, one was excluded until there were no significant correlations remaining. Linear regression analysis with p-value and r-squared is indicated in each panel. All data used to generate plots are available in S1 Data. PAP, population analysis profile. (TIFF) [file pbio.3001346.s002.tiff]

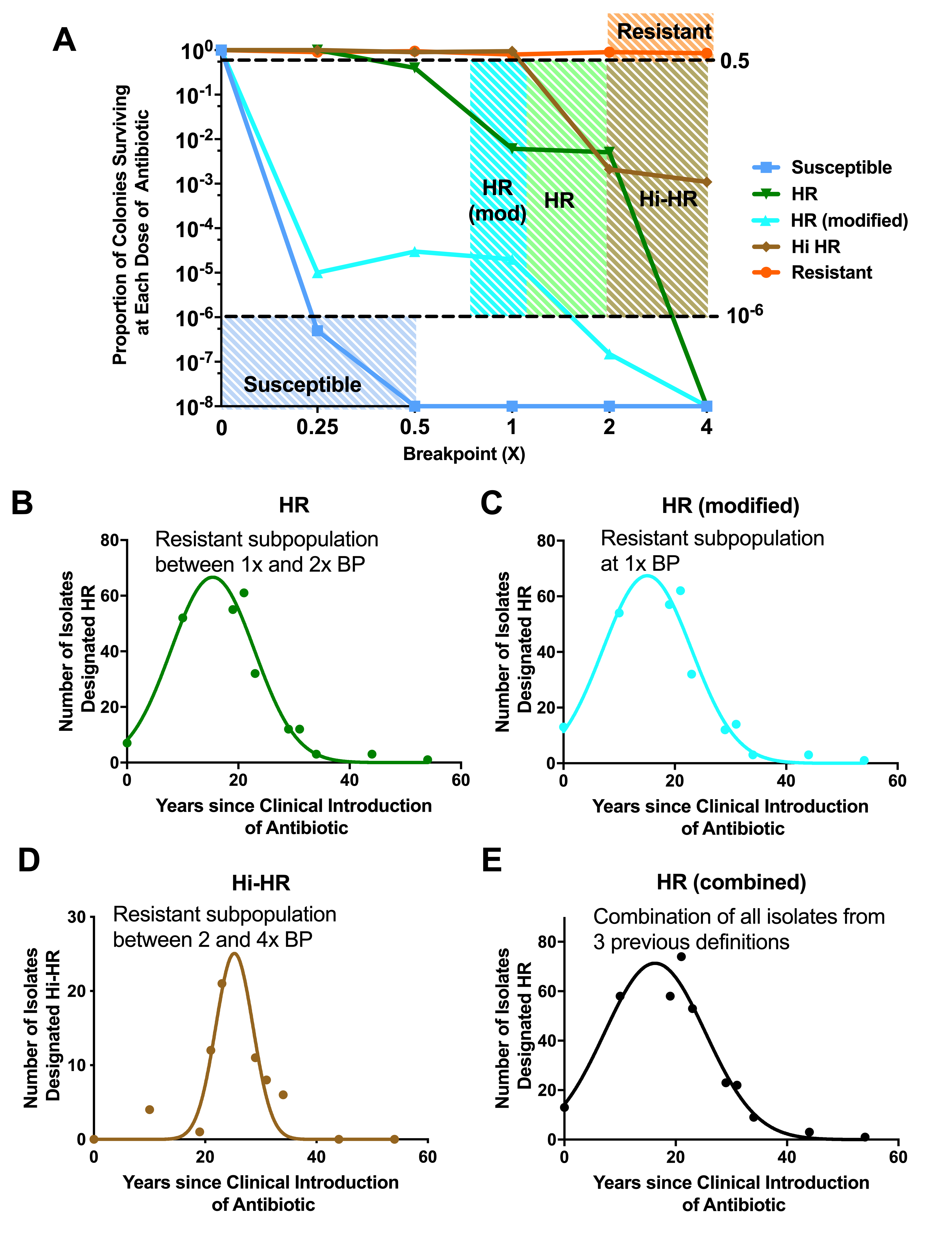

Supplement: S3 Fig — Heteroresistance definition as outlined in S1 Fig was altered to include/exclude distinct resistant subpopulations. (A) A representative graph of a susceptible (blue), HR (green), modified HR (aqua), Hi-HR (brown), and resistant (orange) isolate that fits each definition. Isolates are designated resistant if at 1× or above the breakpoint there is survival of at least 50% (0.5) of the population. If the isolate is not resistant, it will be designated HR if at least 1 in 10−6 bacteria survive at 1× and 2× above the breakpoint. If the isolate is neither resistant nor HR, it will be considered Hi-HR if at least 1 in 10−6 bacteria survive at 2× and 4× the breakpoint. In addition, isolates with at least 1 in 10−6 bacteria surviving at 1× the breakpoint with no requirement for subpopulations surviving at higher concentrations fit the definition for “modified HR.” Susceptible isolates will fall below 10−6 survival at or below the breakpoint. (B–E) Number of isolates designated as (B) HR, (C) HR (modified definition), or (D) Hi-HR based on PAP analysis using these new definitions. In (E), isolates that fit any of the 3 HR definitions in B–D were included as HR (combined). A best fit curve was calculated for each using a Gaussian distribution equation. All data used to generate plots are available in S1 Data. BP, breakpoint; HR, heteroresistant. (TIFF) [file pbio.3001346.s003.tiff]
